# Supplementary material for: Community access to rectal artesunate for malaria (CARAMAL): A large-scale observational implementation study in the Democratic Republic of the Congo, Nigeria and Uganda
Source: PLOS Glob Public Health. 2022 Sep 6;2(9):e0000464. doi: 10.1371/journal.pgph.0000464 (PMC10022208; doi:10.1371/journal.pgph.0000464)
Supplement: S1 Text — (DOCX) [file pgph.0000464.s001.docx]

**Supplementary materials**

**Table A in S1 Text: Authors included under the group name "CARAMAL consortium".**

| **Name** | **Title** | **Affiliation** |
| --- | --- | --- |
| Marek Kwiatkowski | PhD | Swiss TPH, Basel, Switzerland |
| Nadja Cereghetti | MSc | Swiss TPH, Basel, Switzerland |
| Aurelio Di Pasquale | PhD | Swiss TPH, Basel, Switzerland |
| Robert Canavan | MA | Swiss TPH, Basel, Switzerland |
| James Okuma | PhD | Swiss TPH, Basel, Switzerland |
| Silvia Schwarte | MSc | World Health Organization, Switzerland |
| Hans Rietveld | BA | Medicines for Malaria Venture, Switzerland |
| Lydia Kabamba | MD | UNICEF Democratic Republic of Congo |
| Francine Kimanuka | MD | UNICEF Democratic Republic of Congo |
| Sylvie Luketa | MD | UNICEF Democratic Republic of Congo |
| Moulaye Sangare | MD | UNICEF Democratic Republic of Congo |
| Tony Byamungu | MD | UNICEF Democratic Republic of Congo |
| Emmanuel Emedo | MD | UNICEF Nigeria |
| Peter Baffoe | MD | UNICEF Nigeria |
| Oluseyi Olosunde | MD | UNICEF Nigeria |
| Halima Abdu | MD | UNICEF Nigeria |
| Linda Nsahtime-Akondeng | MD | UNICEF Nigeria |
| Garba Safiyanu | MD | UNICEF Nigeria |
| Flavia Mpanga | MD | UNICEF Uganda |
| Fred Kagwire | MD | UNICEF Uganda |
| Michael Mugwanya Musiitwa | MD | UNICEF Uganda |
| Joe Collins Opio | MD | UNICEF Uganda |
| Anita Mbadiwe |  | CHAI Nigeria |
| Tayo Olaleye | Dr. | CHAI Nigeria |
| Remilekun Peregrino |  | CHAI Nigeria |
| Abraham Okita | Dr. | CHAI Nigeria |
| Omowunmi Omoniwa | Dr. | CHAI Nigeria |
| Nnenna Ogbulafor | Dr. | CHAI Nigeria |
| Oluseyi Omokore | MD | CHAI Nigeria |
| Chizoba Fashanu | Dr. | CHAI Nigeria |
| Stella N. Nwosu | MD | CHAI Nigeria |
| Carine Olinga |  | CHAI Democratic Republic of Congo |
| Jenny Bokanga |  | CHAI Democratic Republic of Congo |
| Eric Mukomena | PhD | PNLP Democratic Republic of Congo |
| Jean-Claude Tembele | Dr | CHAI Democratic Republic of Congo |
| Albert Kadjunga | Dr | CHAI Democratic Republic of Congo |
| Fatou Mwaluke | MPH | CHAI Democratic Republic of Congo |
| Alex Ogwal |  | CHAI Democratic Republic of Congo |
| Juliet Nakiganda |  | CHAI Democratic Republic of Congo |
| Isaac Okiring |  | CHAI Uganda |
| Maureen Amutuhaire |  | CHAI Uganda |

**Fig A in S1 Text: Detailed study sites maps. Democratic Republic of Congo (a), Nigeria (b) and Uganda (c).**


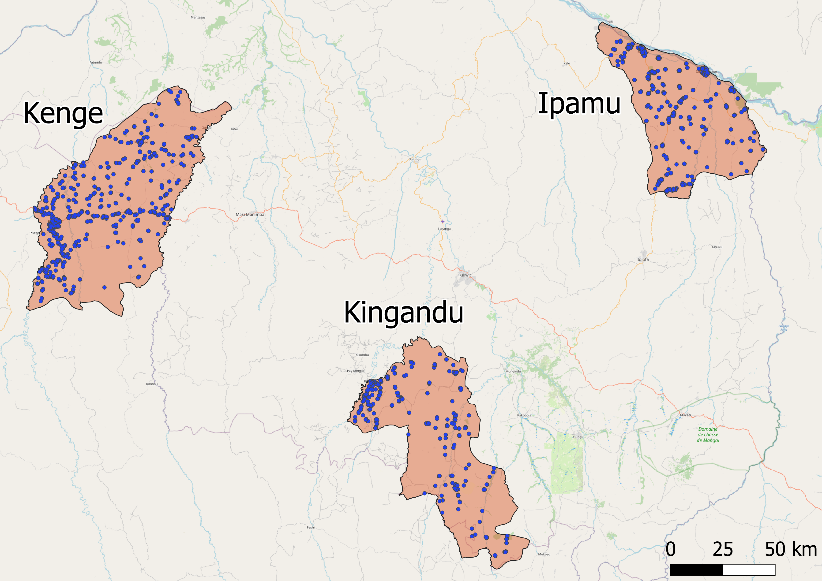


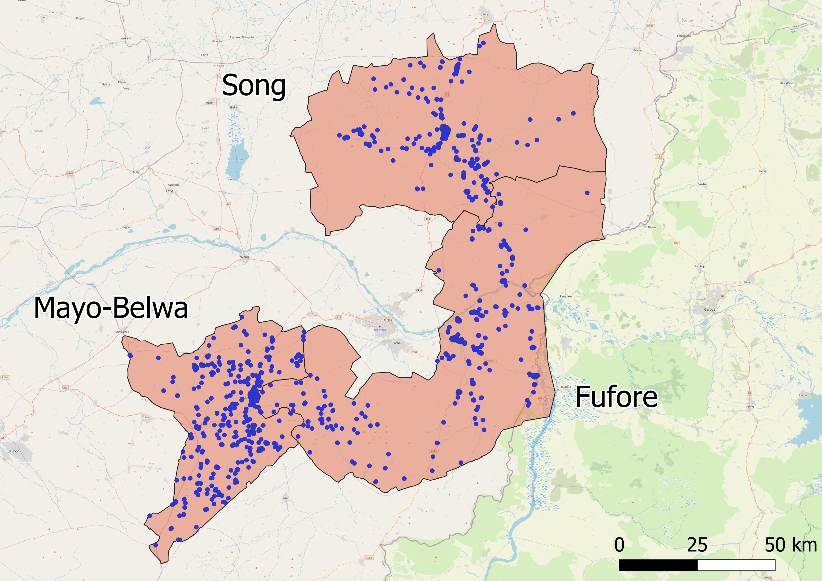


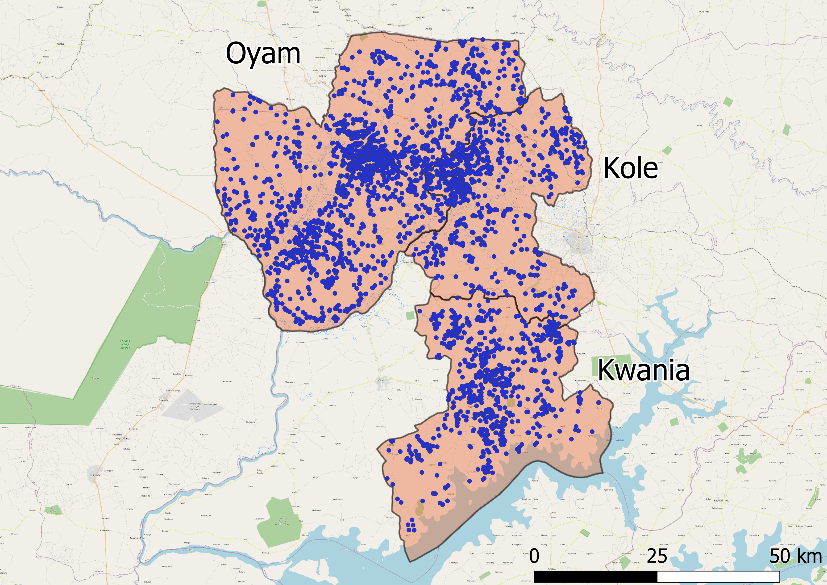


**Table B in S1 Text: Key indicators and survey instruments used in the CARAMAL study, by research question.**

PSS = Patient surveillance system; HCPS = Health care provider survey; HHS = Household survey; EES = Economic evaluation study; HF = Health facility; M&E = routine M & E activity.

| \| **Research questions** \| **Indicator** \| **Source** \| **Indicator description** \| **Frequency** \| \| --- \| --- \| --- \| --- \| --- \| \| **I** \| QA RAS coverage \| M&E, HCPS \| Proportion of trained and functional CHW and primary HF who provide QA RAS \| Annually \| \| RAS availability \| M&E, HCPS \| Proportion of CHW/primary HF with RAS in stock \| Annually \| \| Functional referral facilities \| HCPS \| Proportion of referral health facilities that have the capacity to manage severe malaria in children in line with global guidance \| Annually \| \| Supervision \| M&E, HCPS \| Proportion of CHW/primary HF that received at least one supervisory visit in the past 3 months \| Annually \| \| **I + II** \| Adverse events \| PSS \| Frequency of passively reported adverse events after RAS administration \| Continuous \| \| Delayed haemolytic anaemia \| PSS \| Frequency of delayed haemolytic anaemia within 28 days after RAS administration \| Continuous \| \| **II** \| Treatment seeking \| HHS \| Proportion of children <5 years with a recent history of fever (mild or severe) who attend CHW/primary HF \| Annually \| \| CHW attendance \| PSS \| Proportion of children <5 years with a recent history of fever (mild or severe) who attend CHW/primary HF \| Annually \| \| RAS acceptability among health workers \| HCPS \| Acceptability of pre-referral RAS among health workers \| Annually \| \| RAS acceptability in community \| HHS \| Acceptability of pre-referral RAS among caretakers \| Annually \| \| Referral \| PSS \| Proportion of children <5 years with severe febrile illness seen by CHW/primary HF who completed referral \| Continuous \| \| Direct reporting \| PSS \| Number of children <5 years with severe febrile illness who report directly to RHF \| Continuous \| \| **I + III** \| Pre-referral treatment \| PSS \| Proportion of children <5 years with severe febrile illness managed pre-referral according to guidelines \| Continuous \| \| Post-referral treatment \| PSS \| Proportion of children <5 years with severe febrile illness managed post-referral according to guidelines \| Continuous \| \| **IV** \| Case fatality ratio \| PSS \| Proportion of children <5 years with severe febrile illness seen by CHW/primary HF that resulted in death within 28 days \| Continuous \| \| Malaria infection on day 28 \| PSS \| Proportion of children <5 years with severe febrile illness without parasites on day 28 \| Continuous \| \| Successful referral \| PSS \| Proportion of children <5 years with severe febrile illness seen by CHW/primary HF who completed referral to a referral health facility \| Continuous \| \| **V** \| Costs \| EES \| Total financial cost of managing severe case at community level.  Marginal financial cost of adding RAS to the management. \| Continuous  From project records \| \| Cost-effectiveness \| PSS EES \| Financial cost of RAS interventions per death averted. \| PSS and project records \| \| **Exploratory / contextual** \| Danger signs \| HHS \| Proportion of children <5 years with a recent history of fever who reported danger signs (signs of 'severe febrile illness'). \| Annually \| \| Malaria prevalence \| HHS \| Proportion of children <5 years infected with malaria parasites \| Annually \| |
| --- | --- | --- | --- | --- | --- | --- | --- | --- | --- | --- | --- | --- | --- | --- | --- | --- | --- | --- | --- | --- | --- | --- | --- | --- | --- | --- | --- | --- | --- | --- | --- | --- | --- | --- | --- | --- | --- | --- | --- | --- | --- | --- | --- | --- | --- | --- | --- | --- | --- | --- | --- | --- | --- | --- | --- | --- | --- | --- | --- | --- | --- | --- | --- | --- | --- | --- | --- | --- | --- | --- | --- | --- | --- | --- | --- | --- | --- | --- | --- | --- | --- | --- | --- | --- | --- | --- | --- | --- | --- | --- | --- | --- | --- | --- | --- | --- |

**Table C in S1 Text: Demographic characteristics of study population enrolled in the Patient Surveillance System (PSS), by enrolment location.**

CHW=Community Health Worker. PHC=Primary Health Care (facility). RHF=Referral Health Facility.

|  | **Enrolment CHW/PHC** | | **Enrolment RHF** | |
| --- | --- | --- | --- | --- |
|  | n | % | n | % |
| DR Congo | 3’042 |  | 2’498 |  |
| Health Zone |  |  |  |  |
| Ipamu | 1’128 | 37.1 | 1’163 | 46.6 |
| Kenge | 1’101 | 36.2 | 906 | 36.3 |
| Kingandu | 813 | 26.7 | 429 | 17.2 |
| Sex |  |  |  |  |
| Male | 1’615 | 53.1 | 1’315 | 52.6 |
| Female | 1’427 | 46.9 | 1’183 | 47.4 |
| Age (years) |  |  |  |  |
| 0 - 0.9 | 624 | 20.5 | 507 | 20.3 |
| 1 - 1.9 | 888 | 29.2 | 733 | 29.3 |
| 2 - 2.9 | 647 | 21.3 | 495 | 19.8 |
| 3 - 3.9 | 467 | 15.4 | 417 | 16.7 |
| 4 - 4.9 | 416 | 13.7 | 46 | 13.9 |
|  |  |  |  |  |
| Nigeria | 658 |  | 847 |  |
| LGA |  |  |  |  |
| Fufore | 270 | 41.0 | 313 | 37.0 |
| Mayo Belwa | 271 | 41.2 | 230 | 27.2 |
| Song | 117 | 17.8 | 304 | 35.9 |
| Sex |  |  |  |  |
| Male | 401 | 60.9 | 471 | 55.6 |
| Female | 257 | 39.1 | 376 | 44.4 |
| Age (years) |  |  |  |  |
| 0 - 0.9 | 76 | 11.6 | 94 | 11.1 |
| 1 - 1.9 | 181 | 27.5 | 220 | 26.0 |
| 2 - 2.9 | 186 | 28.3 | 263 | 31.1 |
| 3 - 3.9 | 132 | 20.1 | 162 | 19.1 |
| 4 - 4.9 | 83 | 12.6 | 108 | 12.8 |
|  |  |  |  |  |
| Uganda | 3’927 |  | 2’786 |  |
| District |  |  |  |  |
| Kole | 1’787 | 45.5 | 656 | 23.6 |
| Kwania | 1’055 | 26.9 | 580 | 20.8 |
| Oyam | 1’085 | 27.6 | 1’550 | 55.6 |
| Sex |  |  |  |  |
| Male | 2’093 | 53.3 | 1’553 | 55.7 |
| Female | 1’834 | 46.7 | 1’233 | 44.3 |
| Age (years) |  |  |  |  |
| 0 - 0.9 | 711 | 18.1 | 536 | 19.2 |
| 1 - 1.9 | 1’135 | 28.9 | 830 | 29.8 |
| 2 - 2.9 | 913 | 23.3 | 698 | 25.1 |
| 3 - 3.9 | 729 | 18.6 | 428 | 15.4 |
| 4 - 4.9 | 439 | 11.2 | 294 | 10.6 |
